# WEE1 Inhibition as a Novel Therapeutic Strategy in Cutaneous T-Cell Lymphoma

**DOI:** 10.3390/cancers18172793

**Published:** 2026-08-28

**Authors:** Deniz Özistanbullu, Karola Bahrami, Monika Doll, Gabi Reichenbach, Sarah M. Pöschl, Raphael Wilhelm, Henner Stege, Nadja Zöller, Lars Winkler, Manuel Jäger, Jan P. Nicolay, Sven R. Quist, Roland Kaufmann, Markus Meissner, Bastian Schilling, Johannes Kleemann, Jindrich Cinatl, Stefan Kippenberger

**Affiliations:** 1 Department of Dermatology, Venereology and Allergology, University Hospital Frankfurt, Goethe University, 60590 Frankfurt am Main, Germany; 2 Department of Dermatology, University Medical Center of the Johannes Gutenberg University, 55131 Mainz, Germany; 3 Experimental Pharmacology & Oncology Berlin-Buch GmbH, 13125 Berlin, Germany; 4 Department of Dermatology, Städtisches Klinikum Karlsruhe, 76133 Karlsruhe, Germany; 5 Department of Dermatology, Venereology and Allergology, University Medical Center Mannheim, University of Heidelberg, 68167 Mannheim, Germany; 6 Clinic of Dermatology, Helix Medical Excellence Center Mainz, 55128 Mainz, Germany; 7 Department of Dermatology, Otto-Von-Guericke Universität Magdeburg, 39120 Magdeburg, Germany; 8 Interdisciplinary Laboratory for Pediatric Tumor and Virus Research, Dr. Petra Joh Research Institute, University Hospital Frankfurt, Goethe University, 60590 Frankfurt am Main, Germany

**Keywords:** cutaneous T-cell lymphoma, mycosis fungoides, Sézary syndrome, WEE1, adavosertib, AZD1775, kinase inhibitor screen, cell cycle, DNA damage response, targeted therapy

## Abstract

**Background/Objectives:** Cutaneous T-cell lymphomas (CTCL), most commonly mycosis fungoides and Sézary syndrome, are rare non-Hodgkin lymphomas. Advanced disease responds poorly to current treatments, highlighting the need for new molecularly targeted therapies. WEE1 is a central regulator of the G2/M checkpoint and S-phase progression and has emerged as a therapeutic target in several malignancies, yet it has not been systematically explored in CTCL. **Methods:** We screened a library of more than 2200 kinase inhibitors in CTCL cell lines and selected adavosertib for further study. Its effects were tested in four CTCL lines; primary keratinocytes and fibroblasts; patient-derived malignant CD4^+^ T cells and healthy donor CD4^+^ T cells; and a MyLa xenograft model, using viability, apoptosis, cell-cycle, Western blot, and phospho-protein array assays. **Results:** Adavosertib reduced viability at submicromolar IC_50_ values (0.26–0.56 µM) across all four CTCL lines while largely sparing primary skin cells and was more active in malignant than in healthy donor CD4^+^ T cells. It induced apoptosis and cell-line-specific S-phase and/or G2/M accumulation, lowered WEE1 and phospho-CDK1 (Tyr15), and increased phospho-H2A.X. A phospho-protein array showed activation of checkpoint and stress signalling. In a preliminary xenograft experiment, adavosertib slowed MyLa tumour growth. **Conclusions:** These preclinical data identify WEE1 as a therapeutic target in CTCL and support further preclinical and early-phase clinical evaluation of adavosertib in this disease.

## Simple Summary

Cutaneous T-cell lymphomas are rare cancers that arise from immune cells in the skin. In advanced stages, the disease responds poorly to the available treatments, and patients often run out of effective options. We therefore searched for compounds that could offer a new way to target these cancer cells. From a collection of more than two thousand substances, a drug called adavosertib stood out. It blocks a protein that the cancer cells depend on to divide. In our experiments, adavosertib reduced the survival of lymphoma cells from four cell lines and from patients, while largely sparing normal skin cells. In a preliminary mouse experiment, it also slowed tumour growth in mice. As this drug has already been tested in patients with other types of cancer, our findings provide a concrete rationale to investigate it further in cutaneous T-cell lymphoma and may guide future studies in this disease.

## 1. Introduction

Mycosis fungoides (MF) and Sézary syndrome (SS) are the most common forms of cutaneous T-cell lymphoma (CTCL), a rare and heterogeneous group of primarily cutaneous non-Hodgkin lymphomas arising from the clonal expansion of mature skin-homing T cells [1,2,3]. Early-stage patients may remain stable over long periods, whereas stage IIB to IV disease behaves aggressively, with median survival between one and five years [4].

Several treatment modalities are available for advanced disease, including monoclonal antibodies, immune modulators, HDAC inhibitors, and chemotherapy, but response rates are often low and of limited duration [5,6].

Cure is potentially possible after allogeneic stem-cell transplantation. Yet, this is an approach restricted to a minority of patients because of its toxicity [7]. New targeted approaches are therefore needed for patients with advanced disease [2,3,6,8].

Kinase signalling and cell-cycle control are increasingly recognized as relevant therapeutic targets in CTCL. Several pathways, including JAK/STAT signalling and DNA damage response pathways, have been implicated in CTCL pathogenesis and are being explored therapeutically, but the therapeutic potential of kinase inhibition in CTCL remains largely unexplored [9,10]. We therefore applied a systematic kinase inhibitor screening approach to identify compounds with antitumor activity in CTCL. From this screen, adavosertib was selected as a top candidate for further characterization.

WEE1 is a protein kinase (enzyme) that regulates cell-cycle progression, acting primarily as a molecular “brake” on cell division [11]. WEE1 was originally identified as a protein kinase in the nucleus of the fission yeast *Schizosaccharomyces pombe* [12]. The human orthologue is a nuclear protein of approximately 96 kDa and a crucial factor in regulating cell-cycle progression [13]. WEE1 regulates the G2/M checkpoint by phosphorylating and inhibiting CDK1, thereby preventing mitotic entry in the presence of DNA damage or incomplete DNA replication. Inhibition of WEE1 can force tumour cells to continue cell-cycle progression despite unresolved damage, leading to replication stress, checkpoint failure, and apoptotic cell death. This concept has shown promise across several solid and hematological malignancies [14,15,16]. Adavosertib is the most clinically advanced WEE1 inhibitor and has already been evaluated in early-phase clinical studies in patients with advanced solid tumours, including ovarian cancer and uterine serous carcinoma [17,18,19]. In hematological malignancies, preclinical work has further supported WEE1 as a relevant therapeutic target, including recent data in mature T-cell leukemias and lymphomas [20]. However, WEE1 inhibition has not yet been systematically explored in CTCL.

In the present study, we investigated the activity, selectivity, and mechanism of action of adavosertib in CTCL cell lines, patient-derived malignant T cells, and an in vivo xenograft model.

## 2. Materials and Methods

### 2.1. Cell Culture and Reagents

HuT78 (SS) was obtained from Michael U. Martin (Justus Liebig University Gießen, Gießen, Germany). SeAx (SS) and HH (MF) came from Jan Nicolay (University of Heidelberg, Heidelberg, Germany), and MyLa (MF) from Jean-Philippe Merilo and Edith Chevret (University of Bordeaux, Bordeaux, France). Cell line identity was confirmed by short tandem repeat profiling of HuT78, SeAx, HH, and MyLa. All cell lines were routinely tested for mycoplasma contamination. Culture medium for these four cell lines was RPMI 1640 medium without L-glutamine, to which 1% L-glutamine, 1% penicillin/streptomycin and 7.5% fetal calf serum (Gibco/Thermo Fisher, Waltham, MA, USA) were added. Primary human keratinocytes and fibroblasts were isolated from donor infant foreskins. Keratinocytes were maintained in DermaLife K medium (CellSystems, Troisdorf, Germany). Fibroblasts were cultured in high-glucose Dulbecco’s modified Eagle medium (Gibco/Thermo Fisher) supplemented with 1% penicillin/streptomycin, 1 mM ascorbic acid 2-phosphate (Sigma-Aldrich, St. Louis, MO, USA) and 5% FCS. Cell cultures were kept at 37 °C under 5% CO_2_ in a humidified incubator. Adavosertib was purchased from MedChemExpress (Monmouth Junction, NJ, USA).

### 2.2. Patient Samples

Patients with CTCL were recruited at the University Hospital Frankfurt and the Municipal Hospital Karlsruhe. Donated blood was collected in lithium heparin tubes. Written informed consent was obtained from every participant. Sample collection had been approved by the Ethics Committee of the Faculty of Medicine at the University Hospital Frankfurt and the Medical Faculty Mannheim.

Disease was confirmed clinically and histopathologically in all Sézary syndrome patients. Diagnosis and staging were performed according to ISCL/EORTC criteria. Criteria for peripheral blood involvement were clonality of the T-cell receptor gene, a confirmed aberrant CD4^+^ T-cell population and a raised CD4:CD8 ratio, alongside typical CTCL skin histology and clinical skin findings.

The aberrant T-cell phenotype was defined using CD7 and, where available, CD26. CD26 was not yet part of the routine diagnostic panel in Frankfurt during earlier sampling, so no clinical CD26 status is available for two patients with a CD3^+^CD4^+^CD7^−^ phenotype. CD26 was later established and was available for one Frankfurt patient with a CD3^+^CD4^+^CD7^+^CD26^−^ phenotype. The patient from the Municipal Hospital Karlsruhe had a CD3^+^CD4^+^CD7^−^CD26^−^ phenotype with both markers documented.

Ficoll density-gradient centrifugation was performed to isolate peripheral blood mononuclear cells (PBMCs) from Sézary syndrome patients and healthy donors. CD4^+^ T cells were then obtained from these by negative selection with the MACS CD4^+^ T-Cell Isolation Kit (Miltenyi Biotec, Bergisch Gladbach, Germany), following the manufacturer’s instructions.

For the functional assays, the malignant population was further enriched by magnetic depletion of the aberrantly absent marker, using CD7 MicroBeads to deplete CD7-expressing cells in the CD7^−^ cases and CD26 MicroBeads to deplete CD26-expressing cells in the CD7^+^CD26^−^ case (Miltenyi Biotec). We confirmed the aberrant Sézary phenotype by flow cytometry using CD7 and CD26 on aliquots of the CD4^+^-enriched PBMCs and the negatively selected cells. Before culture, isolated cells were activated for 48 h with TransAct (1:100, Miltenyi Biotec). The medium was RPMI 1640 containing heat-inactivated FBS at 10%, penicillin at 100 U/mL, streptomycin at 100 µg/mL and glutamine at 2 mM. Four cytokines were added, all from Miltenyi Biotec: IL-2, IL-13 and IL-15 at 10 ng/mL and IL-7 at 5 ng/mL. Cultures were kept at 37 °C under 5% CO_2_ in a humidified atmosphere.

### 2.3. Kinase Inhibitor Library Screen

The MCE Kinase Inhibitor Library (HY-L009, MedChemExpress, Monmouth Junction, NJ, USA), comprising approximately 2200 compounds, was screened in a stepwise pipeline in the CTCL cell lines HuT78 and MyLa to identify compounds with antiproliferative activity while limiting nonspecific cytotoxicity. Cells were seeded into 96-well plates and treated with individual compounds at 1 µM and 10 µM. After 48 h of exposure, cell viability was quantified using an MTS assay, and cytotoxicity was assessed in parallel by an LDH release assay (cytotoxicity detection kit (Roche, Mannheim, Germany)). Values for both readouts were normalized to vehicle controls (DMSO) and expressed as a percentage of control. In the primary screen, each compound was tested once at each concentration. Scatter plots were generated using Python (version 3.12) with the matplotlib (version 3.10) and pandas (version 3.0) libraries. Code was developed with the assistance of the AI language model Claude (Anthropic PBC, San Francisco, CA, USA).

### 2.4. Cell Viability (MTS Assay)

Viability was assessed with the CellTiter 96^®^ AQueous One Solution Cell Proliferation Kit (Promega, Madison, WI, USA). We seeded 2 × 10^4^ cells per well in 96-well plates and let them settle overnight before adding DMSO vehicle or varying concentrations of adavosertib. MTS [3-(4,5-dimethylthiazol-2-yl)-5-(3-carboxymethoxyphenyl)-2-(4-sulfophenyl)-2H-tetrazolium] reagent was then added, followed by 1–5 h at 37 °C. Readings were performed using an Asys Expert 96 microplate reader (Deelux Labortechnik GmbH, Gödenstorf, Germany). IC_50_ values were derived from the dose–response curves in GraphPad Prism (Version 10.5.0).

### 2.5. Annexin V/7-AAD Apoptosis Assay

For apoptosis analysis, HuT and MyLa cells were plated at 2.5 × 10^5^ cells per well and SeAx and HH cells at 5 × 10^5^ cells per well. Adavosertib was added at 2.5 µM for 48 h, with DMSO serving as vehicle control. After cells had been harvested and taken up in Annexin V binding buffer, staining was performed with Annexin V–APC and propidium iodide (Miltenyi Biotec, Bergisch Gladbach, Germany) for 20 min in the dark at room temperature. Samples were then diluted with binding buffer and measured by flow cytometry.

### 2.6. Cell-Cycle Analysis

CTCL cells were seeded in 12-well plates and treated with either vehicle control or 2.5 µM adavosertib for 24 h. HuT78 and MyLa cells were seeded at 2.5 × 10^5^ cells per well, whereas SeAx and HH cells were seeded at 5 × 10^5^ cells per well. Treatment was followed by harvesting. Fixation followed harvesting and resuspension in PBS, using ice-cold 70% ethanol over 60 min at 4 °C. Cells were then washed and incubated for 30 min at 37 °C with propidium iodide at 50 µg/mL (Sigma, St. Louis, MO, USA) and RNase at 5 µg/mL. Measurement was performed on a fluorescence-activated cell sorter (FACS; Accuri C6 Plus flowcytometer, BD Biosciences, Heidelberg, Germany).

### 2.7. Caspase-3/7 Activity

For Caspase-3/7 activity, we used the Caspase-Glo^®^ 3/7 assay (Promega, Madison, WI, USA) as specified by the manufacturer’s instructions. HuT and MyLa cells were plated at 1 × 10^4^ cells per well, and SeAx and HH cells at 2 × 10^4^ cells per well, in white-bottom 96-well plates, and then left for 24 h at 37 °C under humidified conditions. After treatment with 2.5 µM adavosertib for 24 h, the luminogenic substrate was added. Luminescence signal was determined using a plate-reading luminometer and normalized to DMSO-treated controls.

### 2.8. Western Blotting

Cell lysis was performed by adding RIPA buffer (Sigma-Aldrich, St. Louis, MO, USA) supplemented with protease and phosphatase inhibitors (Roche, Mannheim, Germany). For each sample, 25 µg of protein was combined with SDS sample buffer and run on sodium dodecyl sulphate-polyacrylamide gel electrophoresis (SDS-PAGE) and then transferred onto nitrocellulose membranes. Blocking was performed with 5% non-fat dry milk for 1 h at room temperature.

Membranes were incubated with the primary antibodies overnight at 4 °C, and secondary antibodies were added for one hour at room temperature. For visualization, we used the enhanced chemiluminescence detection systems from Thermo Fisher Scientific (Waltham, MA, USA) or Cyanagen (Bologna, Italy) as specified by the supplier. GAPDH was used as a loading control.

### 2.9. Phospho-Protein Antibody Array

Phosphorylation profiling was carried out on the Cell Signalling Phospho Antibody Array (Full Moon Biosystems, Sunnyvale, CA, USA), which covers 304 highly specific antibodies. MyLa cells were plated at 2.5 × 10^6^ cells per well in 6-well plates and treated with 2.5 µM adavosertib or vehicle for 24 h at 37 °C. Sample processing was performed following the manufacturer’s instructions. Detection relied on IRDye^®^ 800CW Streptavidin (926-32230; 1 mg/mL; LI-COR Biosciences). Scanning was done on a LI-COR imaging system (LI-COR Odyssey DLX; Lincoln, NE, USA) and then analyzed with Image Studio Software (Version 5.2.5). β-actin served as the reference for normalization.

### 2.10. In Vivo Xenograft Model

All animal experiments complied with the German Animal Welfare Act and had been approved under permit number E0023/23 by the responsible authority (Landesamt für Gesundheit und Soziales, LaGeSo, Berlin, Germany). Female NOG-F mice aged 6 to 8 weeks (Taconic, Leverkusen, Germany) received subcutaneous injections of 3 × 10^5^ MyLa cells.

Two experimental groups were established (*n* = 9 mice per group) and treatment was initiated on day 1 after tumour cell inoculation. Compounds were administered orally once daily on a five-days-on/two-days-off schedule. Control mice received oral vehicle (5% DMSO in 30% *w*/*v* hydroxypropyl-β-cyclodextrin aqueous solution), and the treatment group received 30 mg/kg adavosertib. Tumour volume was measured by calliper three times per week and calculated using the formula V = (length × width^2^)/2. Body weight was monitored in parallel, with the frequency increased to daily measurements during periods of rapid tumour growth. Mice were euthanized when tumour volume reached 1.5 cm^3^ or upon occurrence of critical health conditions.

### 2.11. Statistical Analyses

All statistical analyses were carried out using GraphPad Prism 10 (GraphPad Software, San Diego, CA, USA). For two-group comparisons in vitro, covering apoptosis, caspase-3/7 activity, cell-cycle distribution, and AUC values from patient-derived cells, two-tailed unpaired *t*-tests were applied. For comparisons of cell viability across multiple compound concentrations, ordinary one-way ANOVA with Dunnett’s multiple comparisons test was used. For the in vivo xenograft experiment, tumour volume at the experimental endpoint and AUC values were compared using one-tailed unpaired *t*-tests with Welch’s correction. This was based on the a priori hypothesis of reduced tumour growth under adavosertib treatment, established from the in vitro data. Tumour growth over time was analyzed by two-way repeated-measures ANOVA. Data are shown as mean ± SD, and statistical significance was defined as *p* < 0.05.

## 3. Results

### 3.1. Kinase Inhibitor Screen Identifies Adavosertib as a Top Candidate in CTCL

**Figure 1:**
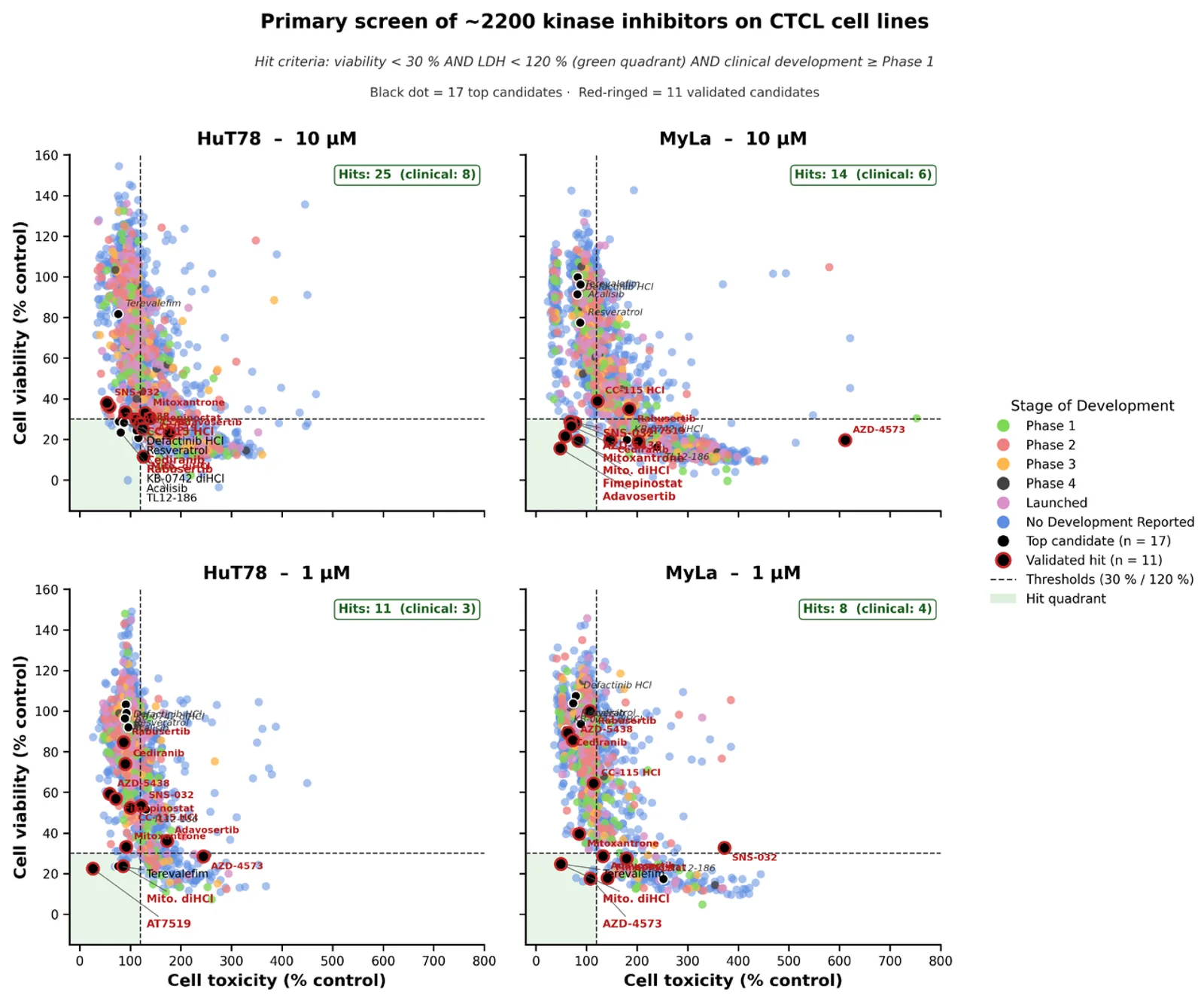
Primary kinase inhibitor screen identifies adavosertib as a top candidate in CTCL. Scatter plots showing cell viability (MTS assay, y-axis) versus cell toxicity (LDH release assay, x-axis) for approximately 2200 compounds from the MCE Kinase Inhibitor Library (HY-L009) tested at 10 µM (**top**) and 1 µM (**bottom**) in HuT78 (**left**) and MyLa (**right**) CTCL cell lines. Each dot represents one compound, colour-coded by clinical development stage. Black dots indicate the 17 top candidates fulfilling predefined hit criteria (viability < 30% and LDH < 120% of control; clinical development ≥ Phase 1); and red-ringed black dots indicate the 11 validated hits confirmed in three independent experiments. Dashed lines mark the viability and toxicity thresholds, and the green-shaded quadrant defines the hit zone. Selected compounds are labelled, and adavosertib was selected as the top candidate for further characterization. Hit counts and the number with reported clinical development are shown in the upper-right corner of each panel.

To identify clinically relevant kinase inhibitors with antiproliferative activity in CTCL, we screened the MCE Kinase Inhibitor Library HY L009 comprising approximately 2200 compounds in a stepwise approach. We used MTS and LDH assays with doses at 1 µM and 10 µM in HuT78 and MyLa cells. Our three predefined selection criteria were viability below 30% of control in the MTS assay, LDH below 120% of control and at least phase 1 clinical development. Criteria were evaluated independently in each of the four screening conditions. Meeting all three criteria in at least one of these four conditions qualified a compound as a candidate hit. The top 17 candidates were identified, of which 11 were confirmed as validated hits upon retesting in three independent experiments. From this set, adavosertib was selected for further investigation (Figure 1). This selection was based on the screening results together with convincing published data for WEE1 inhibition in lymphomas. The complete primary screening dataset is provided in Supplementary Data S1.

### 3.2. Adavosertib Reduces CTCL Cell Viability While Sparing Non-Malignant Skin Cells

**Figure 2:**
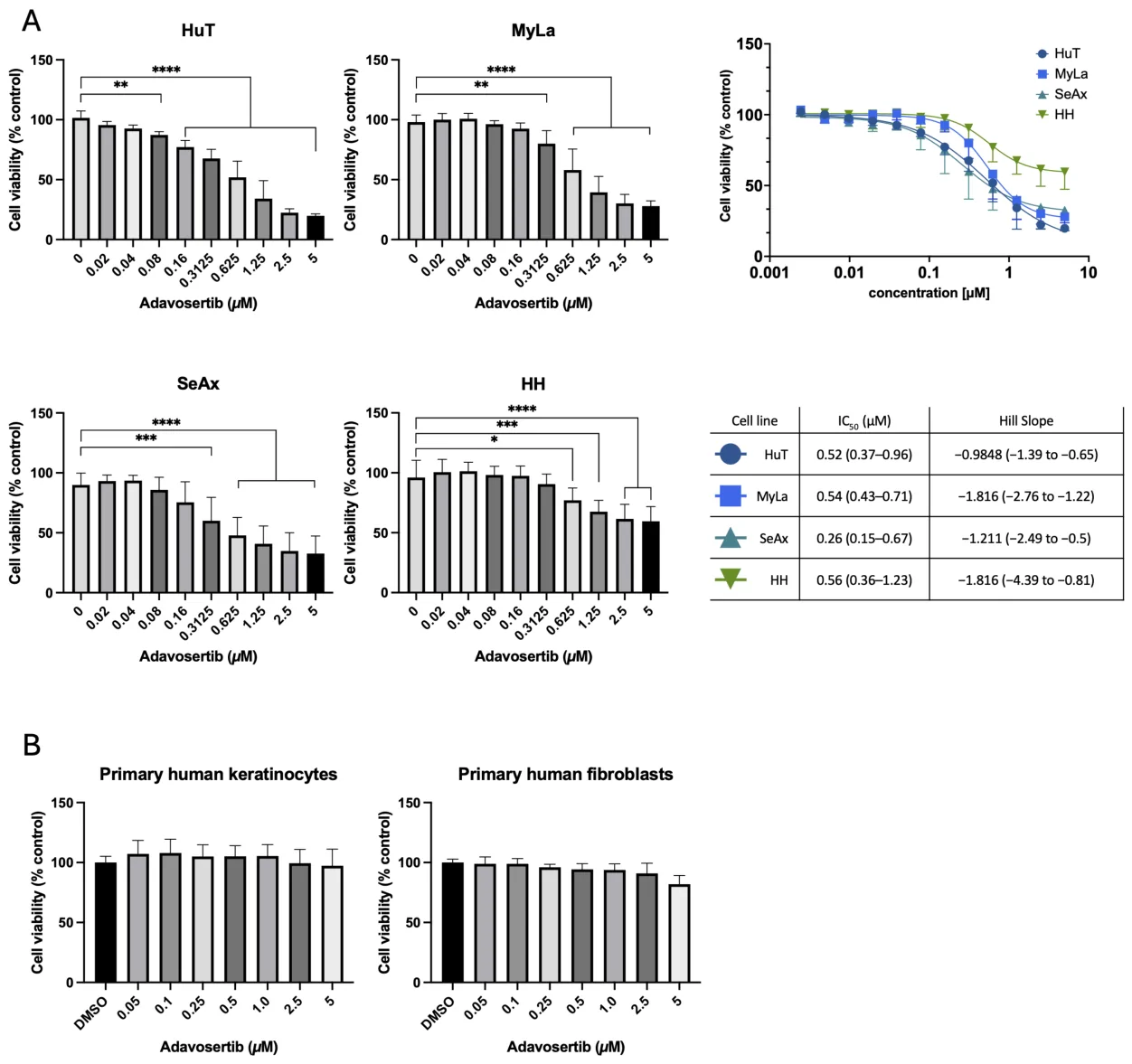
Adavosertib reduces cell viability in CTCL cells while sparing noncancerous skin cells. (**A**) Antiproliferative activity of adavosertib in CTCL cells was assessed by MTS assay and compared to vehicle control. Cells received increasing adavosertib concentrations over 48 h. Values are given relative to DMSO-treated cells (untreated control) and represent means with SD from at least *n* = 3 independent experiments. IC_50_ values (half-maximal inhibitory concentration) were derived from the resulting dose–response curves. (**B**) Primary human keratinocytes and fibroblasts were analyzed with the same approach, again with 48 h of exposure to increasing concentrations and normalization to DMSO-treated cells. Data represent means ± SD from at least *n* = 3 independent experiments. * *p* < 0.05, ** *p* < 0.01, *** *p* < 0.001, **** *p* < 0.0001.

We next assessed the effect of adavosertib on CTCL viability using MTS assays after 48 h of treatment with increasing concentrations. Adavosertib significantly decreased viability in all four established CTCL cell lines (HuT78, MyLa, SeAx, and HH) in a dose-dependent manner (Figure 2A). Nonlinear regression revealed submicromolar IC50 values across the panel, with HuT78 0.52 µM, MyLa 0.54 µM, SeAx 0.26 µM, and HH 0.56 µM, indicating broadly comparable sensitivity with slightly higher potency in SeAx (Figure 2A). In contrast, the viability of primary human fibroblasts and primary human keratinocytes was not affected across the same concentration range, suggesting that adavosertib has tumour cell-specific activity (Figure 2B).

### 3.3. Adavosertib Shows Higher Activity in Patient-Derived Malignant Cells than in Healthy CD4^+^ T Cells

To assess the translational relevance of our findings, we treated malignant CD4^+^ T cells from four Sézary syndrome patients, enriched for the aberrant CD7^−^ or CD26^−^ fraction, and CD4^+^ T cells from the peripheral blood of four healthy volunteers (*n* = 4) with a concentration range of adavosertib over 48 h. Viability was measured by MTS assay and referenced to DMSO controls.

**Figure 3:**
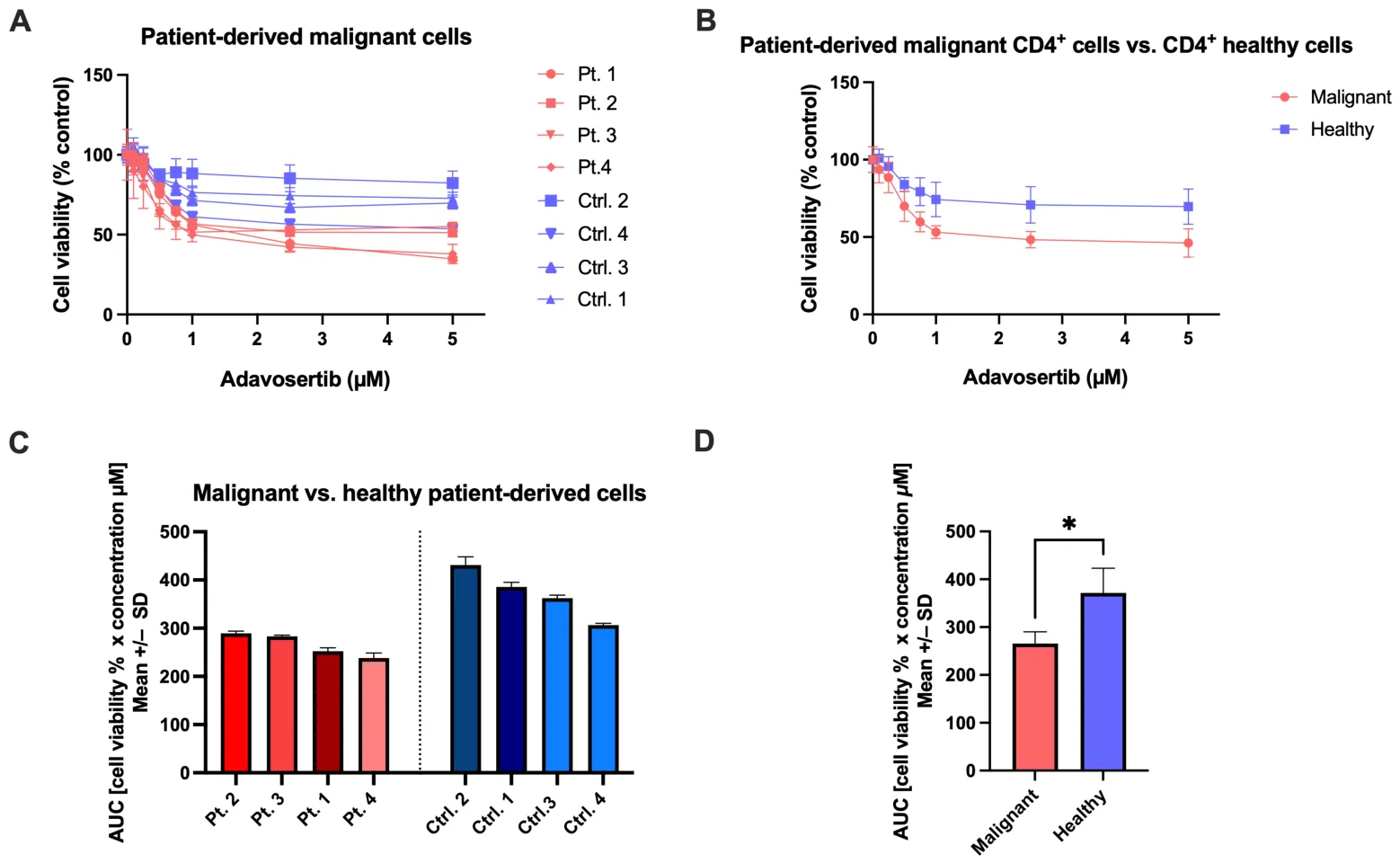
Malignant CD4^+^ T cells respond more strongly to adavosertib than CD4^+^ T cells from healthy donors. Malignant CD4^+^CD7^−^ T cells from four Sézary syndrome patients and peripheral blood CD4^+^ T cells from four healthy donors received a concentration range of adavosertib over 48 h. Viability was read out by MTS assay and referenced to DMSO-treated controls. (**A**) Individual dose–response curves for all donors. (**B**) Mean dose–response curves for malignant and healthy CD4^+^ T cells. (**C**) Area under the curve (AUC) calculated for each individual donor to quantify overall sensitivity to adavosertib across the tested concentration range. (**D**) Comparison of mean AUC values between malignant and healthy groups. Data are shown as mean ± SD. * *p* < 0.05.

Adavosertib reduced viability of malignant cells in a concentration-dependent manner, with noticeable inter-donor variability (Figure 3A). In contrast, viability of healthy donor CD4^+^ T cells was less affected across the tested concentration range (Figure 3A,B). To compare overall drug sensitivity between groups, we calculated the area under the curve (AUC) for each individual dose–response profile. Malignant samples showed lower AUC values than healthy controls, and the mean AUC was significantly reduced in the malignant group (Figure 3C,D). Clinical characteristics of the patient cohort are provided in Supplementary Table S1.

### 3.4. Adavosertib Triggers Apoptosis and Alters Cell-Cycle Progression in CTCL Cells

**Figure 4:**
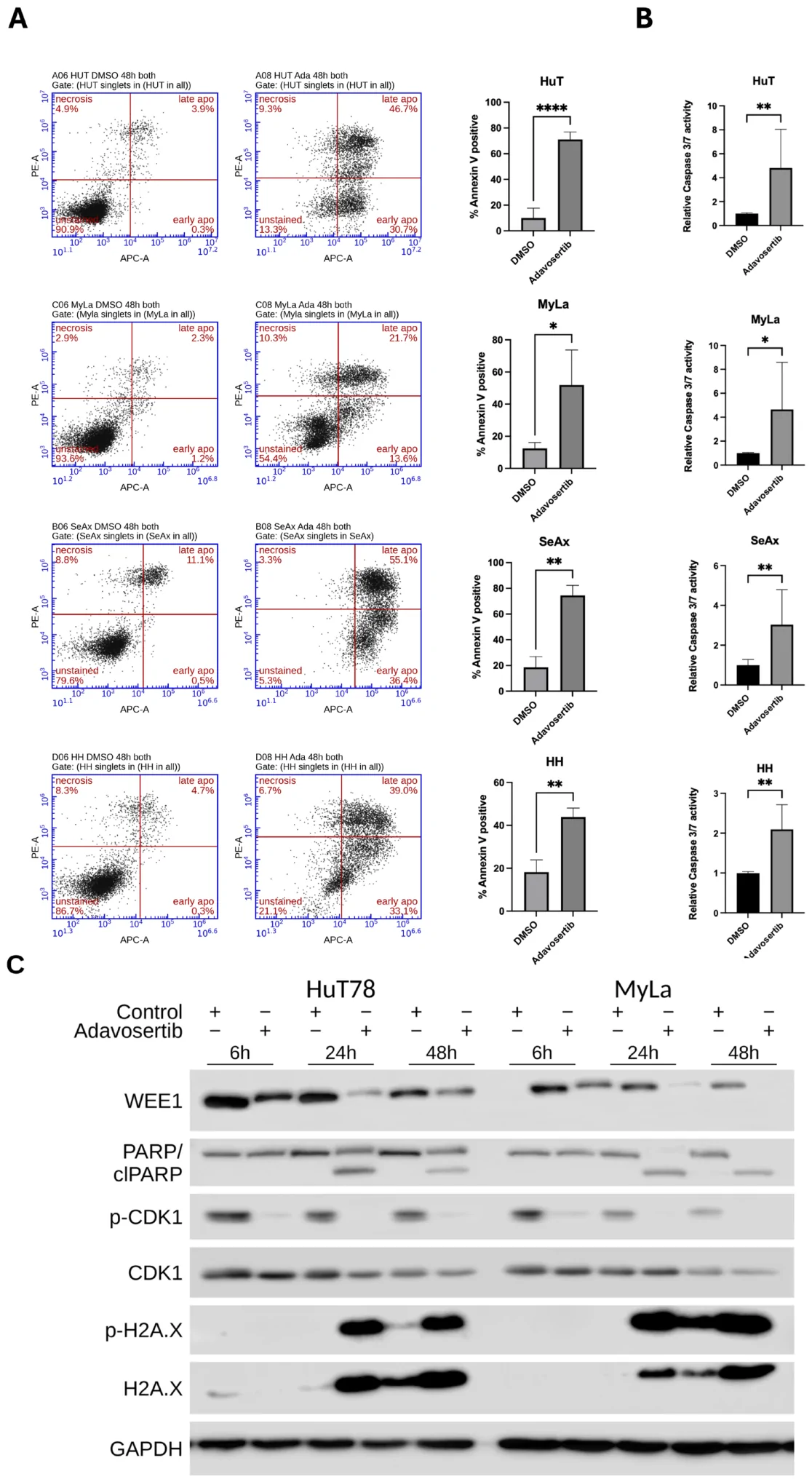
Adavosertib triggers apoptosis in CTCL cells. (**A**) After 48 h of treatment with 2.5 µM adavosertib or DMSO, cells were stained for 20 min with Annexin V–FITC and 7-AAD, and the proportion of Annexin V-positive cells was measured by flow cytometry. The left side shows representative data and the right side depicts the mean values with SD from *n* = 3 independent experiments. (**B**) Caspase-3/7 activation was measured using the Caspase-Glo 3/7 assay after 24 h treatment with 2.5 µM adavosertib. Caspase activity was normalized to cells treated with DMSO (untreated control). Mean values of *n* = 3 independent experiments with SD are plotted. * *p* < 0.05, ** *p* < 0.01, **** *p* < 0.0001. HuT and MyLa cells were treated with DMSO or 2.5 µM adavosertib for the indicated time periods. (**C**) Protein expression levels were assessed by Western blot analysis, with GAPDH serving as a loading control. One representative blot of three independent experiments is shown. Imaging was digital, with brightness and contrast adjusted for each blot globally to improve clarity. No individual lane was changed or removed.

Based on the significant reduction in cell viability, we next analyzed apoptotic cell death by Annexin V/7-AAD staining, which showed apoptosis induction after 48 h at 2.5 µM adavosertib treatment. All four cell lines, HuT78, MyLa, SeAx, and HH, showed significantly more Annexin V-positive cells than the respective control (Figure 4A). This was confirmed by significantly increased caspase-3/7 activity in every cell line at 24 h (Figure 4B).

**Figure 5:**
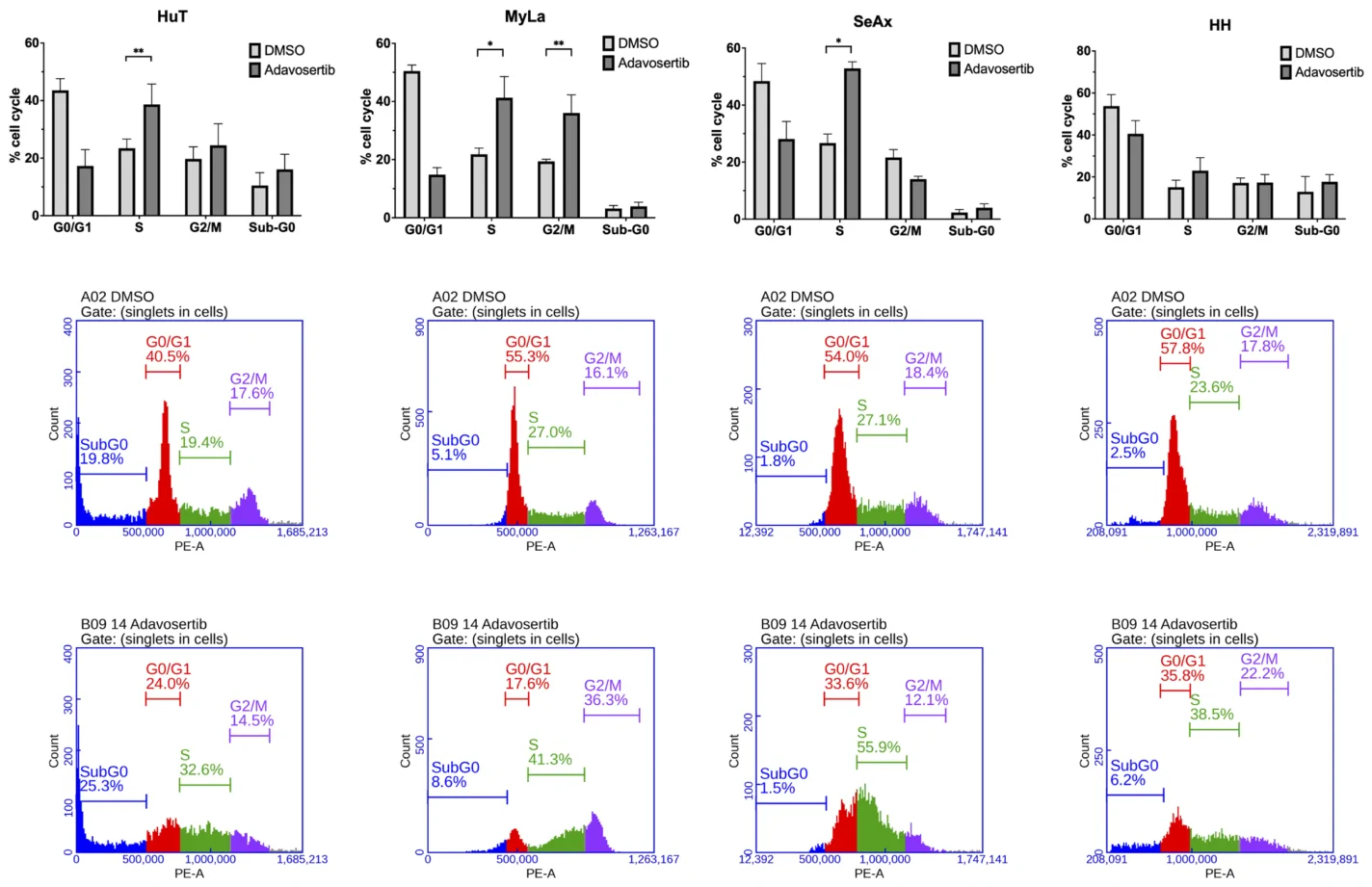
Adavosertib induces cell-line-dependent changes in cell-cycle distribution in CTCL cells. After 24 h treatment with vehicle or 2.5 µM adavosertib, the four cell lines were fixed in 70% ethanol and stained with propidium iodide (PI). Flow cytometry was used to determine the cell-cycle distribution. Bar graphs show mean values ± SD of *n* = 3 independent experiments. Representative PI histograms from one experiment are shown below each bar graph. * *p* < 0.05, ** *p* < 0.01.

Given the known role of WEE1 inhibition in cell-cycle control, we assessed whether adavosertib affects cell-cycle distribution. Flow-cytometric analysis of PI-stained CTCL cells was performed after 24 h of 2.5 µM adavosertib. Adavosertib induced clear cell-line-specific shifts in cell-cycle profiles (Figure 5). In HuT78 and MyLa cells, treatment led to a pronounced accumulation of cells in S phase compared with vehicle-treated controls. In SeAx cells, adavosertib increased the proportion of cells in G2/M. HH cells showed a mixed pattern, with enrichment in S phase together with a relative increase in G2/M (Figure 5).

Western blot analyses were performed to investigate molecular changes associated with adavosertib treatment in HuT78 and MyLa cells across three time points (6 h, 24 h, 48 h). Adavosertib reduced WEE1 protein levels in a time-dependent manner in both cell lines. PARP cleavage was detected, indicating induction of apoptosis. Phosphorylation of CDK1 at Tyr15 decreased under adavosertib treatment, most prominently at 24 h and 48 h, while total CDK1 levels remained largely unchanged. Furthermore, phosphorylation of H2A.X (Ser139) and total H2A.X levels were increased under adavosertib treatment, consistent with DNA damage response activation (Figure 4C). Densitometric quantification of all three independent experiments is provided in Supplementary Table S2, and the original uncropped blots are shown in Supplementary File S1.

To obtain exploratory insights into signalling changes associated with adavosertib, we performed a phospho-protein antibody array in MyLa cells after 24 h exposure to 2.5 µM adavosertib (Supplementary Figure S1). Adavosertib increased phosphorylation of checkpoint kinase 2 (Chk2 Thr68), CREB (Ser133), AKT1 (Thr308), and β-catenin (Thr41/Ser45), accompanied by higher signals for proteins linked to cell-cycle control such as CDC25C and Rb (Ser807). In parallel, phosphorylation of several proteins was reduced, including PKCθ (Thr538), p90RSK (Thr359/Ser363), SHP-2 (Tyr542), and FAK (Ser910), together with changes in T-cell signalling-associated kinases such as LYN (Tyr507). Overall, the phospho-proteomic profile is consistent with the activation of checkpoint and stress responses, alongside the attenuation of selected pro-survival signalling pathways, in CTCL cells.

### 3.5. Adavosertib Limits CTCL Tumour Growth in a Preliminary Xenograft Model

Finally, we evaluated adavosertib in vivo using a MyLa xenograft model. Adavosertib-treated mice showed significantly slower tumour growth compared with vehicle controls (Supplementary Figure S2A,B). At day 24 after tumour injection, tumour volumes were significantly reduced in the adavosertib group (Supplementary Figure S2C), and analysis of tumour growth curves by AUC confirmed a significant treatment effect (Supplementary Figure S2D). These data support the in vivo antitumour activity of adavosertib in CTCL.

## 4. Discussion

Our study identifies the WEE1 inhibitor adavosertib as a highly active compound in CTCL models. In a stepwise screen of more than 2000 compounds, adavosertib was selected from the set of validated hits for deeper characterization and consistently reduced viability across multiple established CTCL cell lines at submicromolar concentrations. In contrast, primary human keratinocytes and fibroblasts showed little to no loss of viability across the same concentration range, suggesting a degree of selectivity for malignant cells in vitro. Similar effects were observed in primary samples, where patient-derived malignant T cells were more strongly affected than CD4^+^ T cells from healthy donors.

Adavosertib treatment resulted in pronounced apoptotic responses and cell-cycle alterations in CTCL cells. Annexin V/7-AAD analysis revealed an increased proportion of apoptotic cells across all CTCL cell lines, which was accompanied by enhanced caspase-3/7 activity. Consistent with these findings, PARP cleavage was detected by immunoblotting, supporting activation of caspase-dependent apoptosis.

In addition, adavosertib induced marked, cell-line-specific changes in cell-cycle distribution. HuT78 and MyLa cells accumulated predominantly in S phase after 24 h of treatment, indicating impaired progression through DNA replication. In contrast, SeAx cells showed an increased fraction in G2/M, whereas HH cells displayed a mixed phenotype with enrichment in both S phase and G2/M. Together, these patterns are consistent with the established role of WEE1 in regulating S-phase progression and the G2/M checkpoint, and support a model in which WEE1 inhibition promotes replication stress, DNA damage, and subsequent apoptotic cell death in susceptible CTCL cells [11,14,15,16].

Mechanistically, Western blot analyses provided clear support that adavosertib acts through WEE1 inhibition in CTCL cells. In HuT78 and MyLa cells, WEE1 protein levels decreased after treatment and inhibitory phosphorylation of CDK1 at Tyr15 was reduced, while total CDK1 remained largely unchanged. At the same time, phosphorylation of H2A.X and total H2A.X levels were increased under adavosertib, consistent with replication-associated stress and DNA damage. Overall, these results indicate that adavosertib impairs checkpoint control and contributes to the cell-cycle disturbances and apoptotic responses observed in our models [14,15,16]. The phospho-protein array data also support this model. After adavosertib treatment, phosphorylation of proteins related to checkpoint and stress signalling, including Chk2, CREB, CDC25C, and Rb, was increased, whereas phosphorylation of several proteins associated with proliferation and survival, such as PKCθ, p90RSK, SHP-2, FAK, and LYN, was reduced. This pattern suggests that the effects of adavosertib are not limited to the WEE1–CDK1 axis but involve broader signalling changes. Similar pathway-level effects after WEE1 inhibition have also been described in mature T-cell lymphoma models [16,20].

Our in vitro findings were in line with the reduced tumour growth observed in our preliminary xenograft experiment.

However, individual tumour growth varied between animals, including the vehicle group. This might reflect differences in initial engraftment and normal biological variation between animals. This pattern is consistent with our own prior CTCL xenograft work and with the original description of the MyLa model itself [21,22,23,24]. Despite this variability, the difference between treatment groups was significant over time and at the study endpoint.

Our findings are in line with recent work in related mature T-cell malignancies. Schmidt et al. identified WEE1 inhibition as an effective strategy in T-cell leukemias and lymphomas and also reported selective activity in malignant cells [20]. Although CTCL is a distinct disease entity, these findings indicate that dependence on WEE1-mediated checkpoint control may represent a target in different T-cell neoplasms.

The variability we observed in primary patient samples likely reflects the molecular heterogeneity of CTCL and underscores the need for predictive biomarkers to define which patients are most likely to benefit from WEE1 inhibition [4,10,25]. TP53 status and defects in S-phase are potential determinants to explain differences in sensitivity [25,26].

A further candidate marker is SETD2 status. The loss of SETD2 sensitizes cells to WEE1 inhibition by depleting dNTPs and causing replication stress [27]. SETD2 is a frequently altered gene in mature T-cell lymphomas [28,29]. This would be consistent with our own findings, including increased phospho-H2A.X and increased Chk2 phosphorylation after adavosertib treatment, which are markers of DNA damage and replication stress.

Across our functional assays, HH showed the weakest response of the four cell lines. Differences in regulators like SETD2 might explain this pattern and the differences in response among our primary blood-derived patient samples. Profiling markers such as SETD2 across our cell lines and patient samples is a natural next step to identify which patients are most likely to benefit from WEE1 inhibition.

From a translational point of view, adavosertib is of interest because it has already been evaluated in early-phase clinical trials in solid tumours, which provides some basis for dose finding and safety assessment in future studies [18,19,30]. Combination approaches may also be worth exploring. HDAC inhibitors are approved in CTCL, and WEE1 inhibition has shown activity in combination with belinostat in myeloid malignancies such as AML and MDS [31]. As we could show for the first time that WEE1 inhibition has antitumour activity in CTCL, comparing adavosertib with newer and more selective WEE1 inhibitors such as azenosertib is an important next step [32].

Some limitations of this study should be considered. The primary screen used a single 48 h endpoint. Compounds with delayed mechanisms of action extending beyond 48 h may not have been identified. Future screens should investigate additional time points. The phospho-protein array analysis was exploratory, and the functional relevance of individual signalling changes remains to be clarified. In addition, although our results were consistent across several cell lines and supported by primary samples and xenograft data, CTCL is a biologically heterogeneous disease with recurrent alterations in genes such as TP53, chromatin-modifying genes, CDKN2A/B, and JAK/STAT pathway members. Larger patient cohorts will be needed to define which subgroups are most likely to benefit from WEE1 inhibition. Although the reduction in p-CDK1 (Tyr15) and in WEE1 protein itself supports on-target activity, we did not perform genetic WEE1 knockdown or knockout [33]. Confirming our observed effects with genetic WEE1 depletion remains an important next step for future work. The xenograft model also does not fully reflect the complexity of the human tumour microenvironment. Further, treatment was initiated one day after inoculation, without confirming successful tumour engraftment at that time, which might have contributed to the variability observed between animals. Future studies should therefore validate candidate biomarkers in additional models and patient material and further explore rational combination strategies for adavosertib in CTCL.

## 5. Conclusions

Our data support WEE1 inhibition as a promising strategy in CTCL. Adavosertib showed consistent antiproliferative activity in CTCL cell lines, preferential activity in patient-derived malignant cells compared with healthy CD4^+^ T cells and mechanistic changes consistent with replication stress, checkpoint disruption, and apoptosis. In a preliminary xenograft experiment, adavosertib significantly delayed MyLa xenograft growth. Together with recent translational work in mature T-cell lymphomas, these findings provide a rationale for further preclinical and clinical evaluation of WEE1-targeted approaches in CTCL.

## Data Availability

The raw data supporting the conclusions of this article will be made available by the authors on request.

## Supplementary Materials

The following supporting information can be downloaded at: https://www.mdpi.com/article/10.3390/cancers18172793/s1, Data S1: Primary Kinase Inhibitor Screen in CTCL; Table S1: Clinical and immunophenotypic characteristics of the Sézary syndrome patients included in the ex vivo assays; Table S2: Western blot densitometry; File S1: Original Western blot images for Figure 4C; Figure S1: Phospho-protein antibody array reveals adavosertib-associated signalling changes in CTCL cells; Figure S2: Adavosertib inhibits tumour growth in a CTCL xenograft mouse model.

## Abbreviations

The following abbreviations are used in this manuscript:

7-AAD	7-aminoactinomycin D
ANOVA	analysis of variance
AUC	area under the curve
CDK1	cyclin-dependent kinase 1
CTCL	cutaneous T-cell lymphoma
DMSO	dimethyl sulfoxide
DNA	deoxyribonucleic acid
EORTC	European Organisation for Research and Treatment of Cancer
FBS	fetal bovine serum
FCS	fetal calf serum
HDAC	histone deacetylase
IC_50_	half-maximal inhibitory concentration
ISCL	International Society for Cutaneous Lymphomas
LDH	lactate dehydrogenase
MF	mycosis fungoides
MTS	3-(4,5-dimethylthiazol-2-yl)-5-(3-carboxymethoxyphenyl)-2-(4-sulfophenyl)-2H-tetrazolium
PBMC	peripheral blood mononuclear cell
PI	propidium iodide
SD	standard deviation
SDS-PAGE	sodium dodecyl sulphate-polyacrylamide gel electrophoresis
SS	Sézary syndrome
TCR	T-cell receptor
