# Supplementary material for: WEE1 Inhibition as a Novel Therapeutic Strategy in Cutaneous T-Cell Lymphoma
Source: Cancers (Basel). 2026 Aug 28;18(17):2793. doi: 10.3390/cancers18172793 (PMC13565369; doi:10.3390/cancers18172793)
Supplement: Supplementary file 1 [file cancers-18-02793-s001.zip › Supplementary Figure S1.pdf]

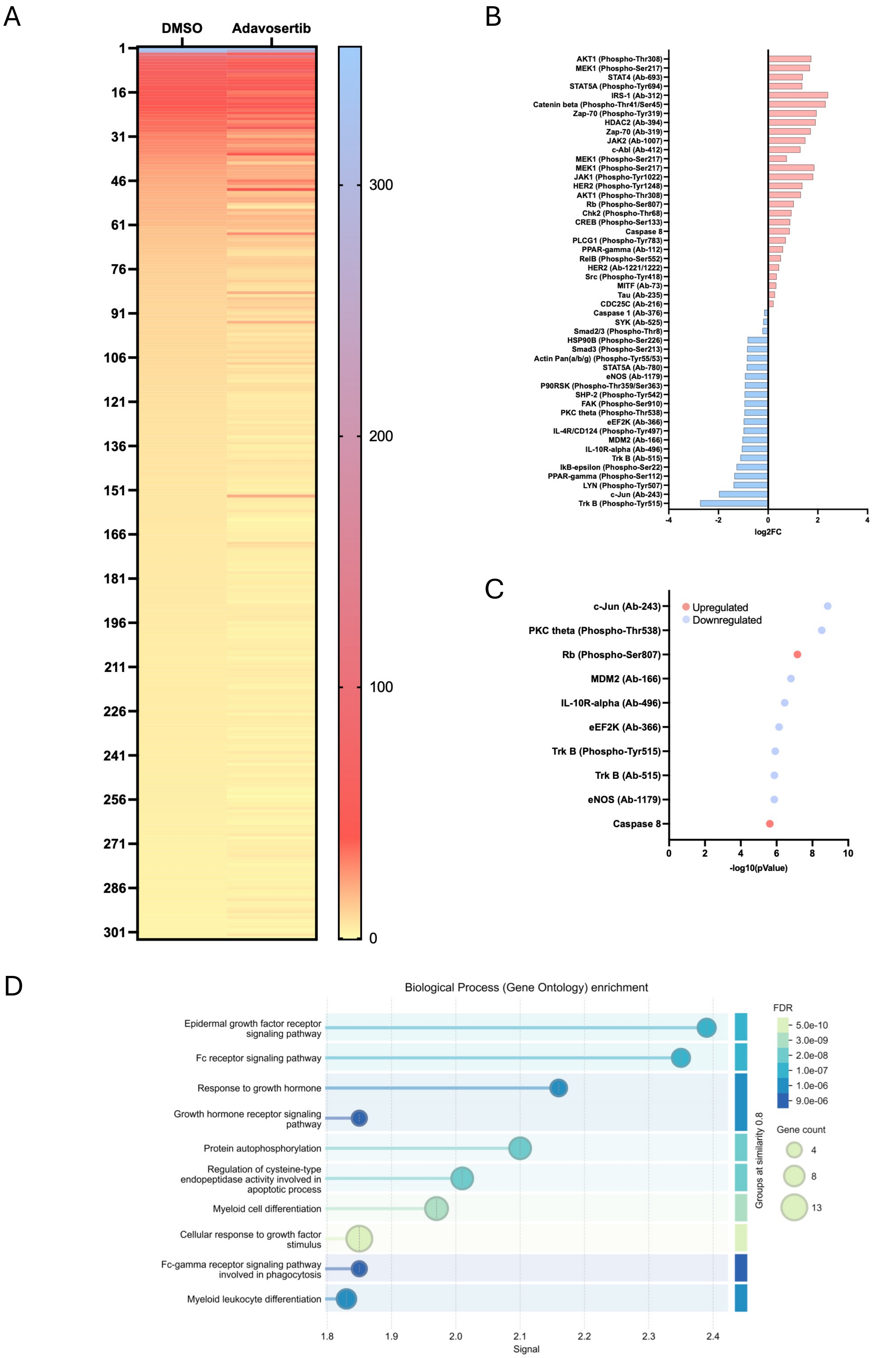

Supplementary Figure S1. Phospho-protein antibody array reveals adavosertib-associated signalling changes in CTCL cells. MyLa cells were treated with vehicle or adavosertib (2.5 μM) for 24 h and protein extracts were analyzed using a phospho-protein antibody array covering 304 targets. **(A)** Heatmap showing phosphorylation signals for all array targets in adavosertib- and vehicle-treated cells.**(B)** Differentially regulated targets defined by an absolute $\log_2$ fold change ≥ 0.14 (~10% change). **(C)** Ranked list of phospho-proteins with the most statistically significant changes. **(D)** Gene Ontology biological process enrichment analysis of differentially regulated targets, showing the top ten enriched processes.
