# Supplementary material for: WEE1 Inhibition as a Novel Therapeutic Strategy in Cutaneous T-Cell Lymphoma
Source: Cancers (Basel). 2026 Aug 28;18(17):2793. doi: 10.3390/cancers18172793 (PMC13565369; doi:10.3390/cancers18172793)
Supplement: Supplementary file 1 [file cancers-18-02793-s001.zip › Supplementary Figure S2.pdf]

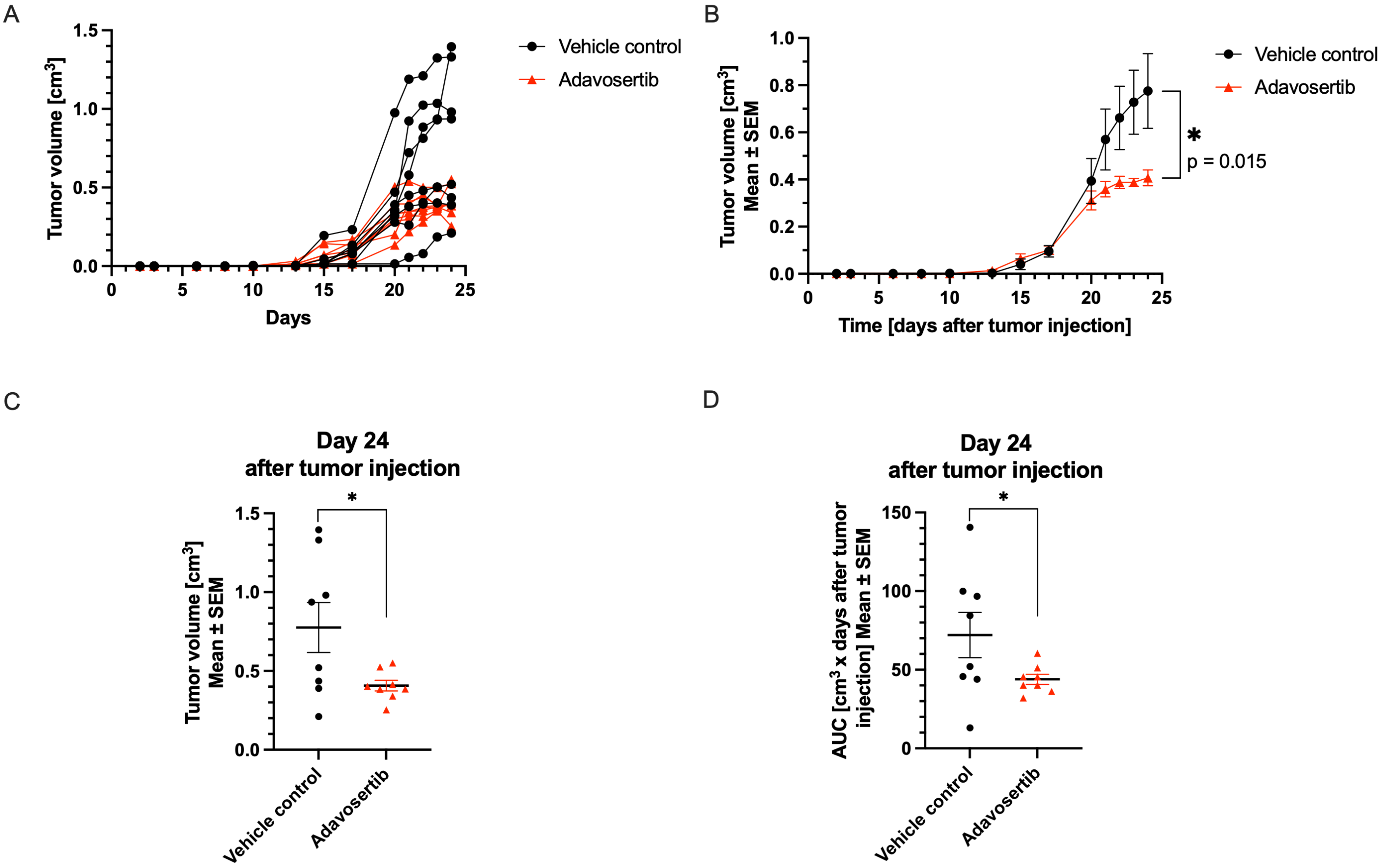

Supplementary Figure S2. Adavosertib inhibits tumor growth in a CTCL xenograft mouse model. injected tumor growth per treatment group. Data are shown as mean ± SD. *p < 0.05. subcutaneously and treated by oral gavage starting on day 1 after cell inoculation with either vehicle or adavosertib (30 mg/kg) once daily on a 5-days-on/2-days-off schedule (n = 9 per group). **(a)** Individual tumor growth curves over time. **(b)** Mean tumor volume ± SD over time. **(c)** Individual and mean tumor volumes at the experimental endpoint. **(d)** Mean area under the curve (AUC) values summarizing overall
