# Supplementary material for: WEE1 Inhibition as a Novel Therapeutic Strategy in Cutaneous T-Cell Lymphoma
Source: Cancers (Basel). 2026 Aug 28;18(17):2793. doi: 10.3390/cancers18172793 (PMC13565369; doi:10.3390/cancers18172793)
Supplement: Supplementary file 1 [file cancers-18-02793-s001.zip › Supplementary File S1.pdf]

Supplementary File S1. Original Western blot images for Figure 4C

Original uncropped scans of the Western blots shown in Figure 4C. On each blot, HuT78 cells are shown on the left and MyLa cells on the right, with the three time points (6 h, 24 h, 48 h) and both treatment conditions (DMSO, adavosertib 2.5 µM) as labeled. GAPDH was used as loading control. Molecular weight markers are shown at the left margin. Blots were imaged digitally and brightness and contrast were adjusted globally for each blot; no individual lanes were modified or removed.

Note on ladder visibility: Because multiple target proteins were probed from the same transfer, membranes were cut into strips by expected protein size, guided by the ladder, before antibody incubation. Each blot below therefore shows only the ladder bands on its own strip, not the full ladder. A single GAPDH loading-control strip is in some cases shared between two target-protein sections probed from the same membrane (e.g., WEE1/H2A.X and phospho-H2A.X on 25-09-09; PARP/cleaved PARP and Cdc2/phospho-cdc2 on 25-09-16).

WEE1 (with GAPDH loading control)

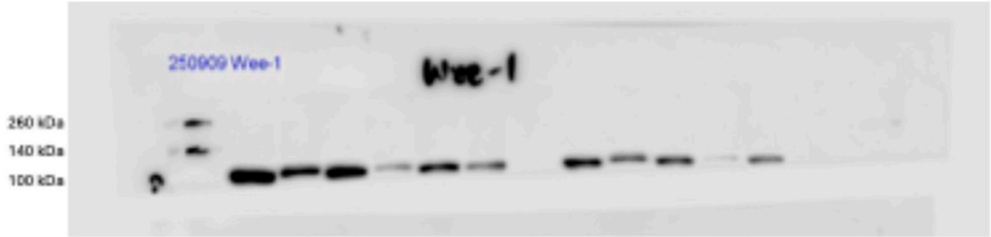

WEE1, molecular weight: 96 kDa. Experiment 25-09-09.

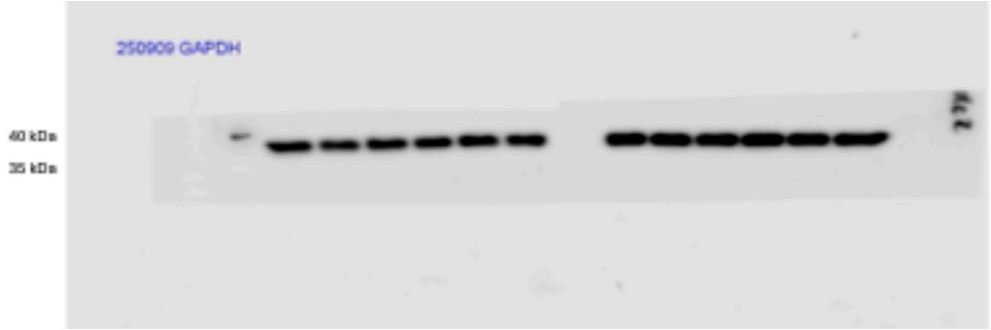

GAPDH loading control, molecular weight: 37 kDa (same samples, 25-09-09).

PARP / cleaved PARP (with GAPDH loading control)

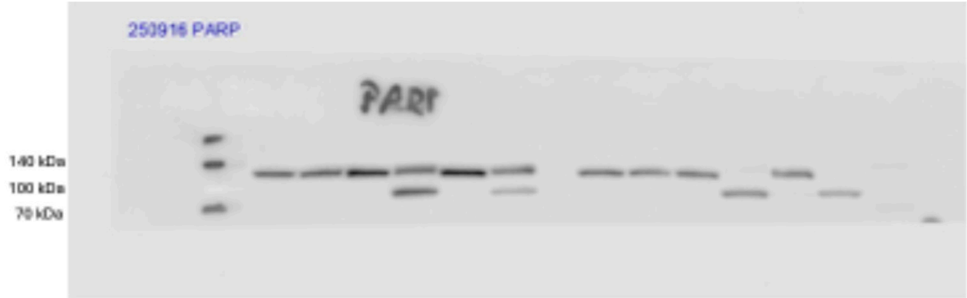

PARP, molecular weight: 116 kDa; cleaved PARP, molecular weight: 89 kDa. Experiment 25-09-16.

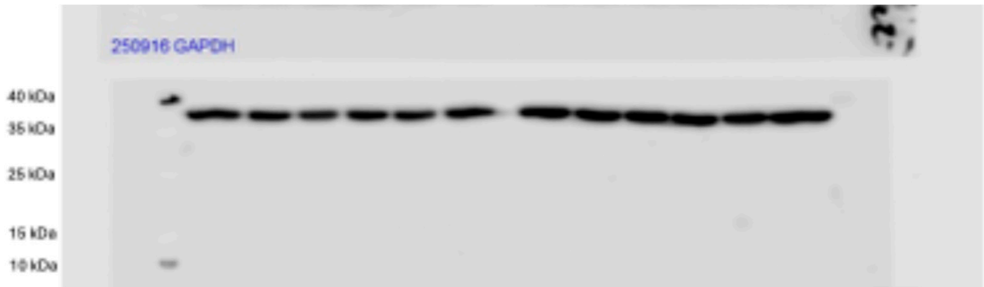

GAPDH loading control, molecular weight: 37 kDa (same samples, 25-09-16).

Cdc2 and phospho-cdc2 (Tyr15) (with GAPDH loading control)

Note: The protein detected on this blot is labeled "cdc2" / "phospho-cdc2" on the original raw image; it is referred to as CDK1 / p-CDK1 in the main-text Figure 4C. Cdc2 (CDC2) and CDK1 are synonyms for the same gene product (current HGNC symbol: CDK1), and "phospho-cdc2 (Tyr15)" and "p-CDK1 (Tyr15)" refer to the identical inhibitory phosphorylation site. Both names are used interchangeably in the literature.

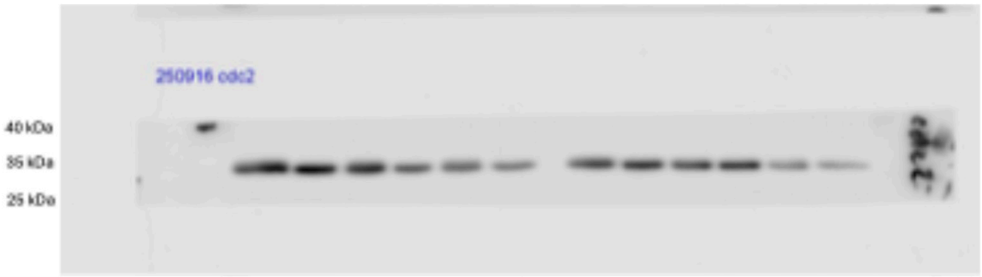

Cdc2, molecular weight: 34 kDa. Experiment 25-09-16.

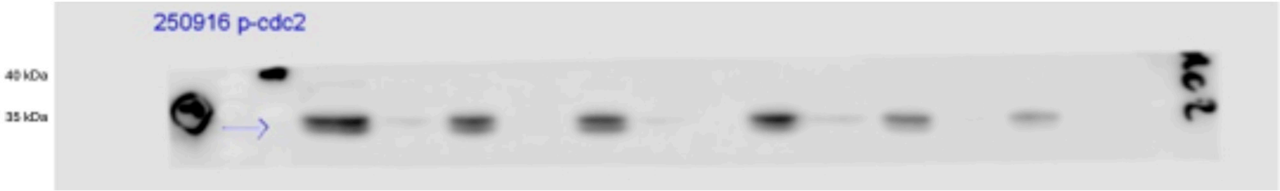

phospho-cdc2 (Tyr15), molecular weight: 34 kDa. Experiment 25-09-16.

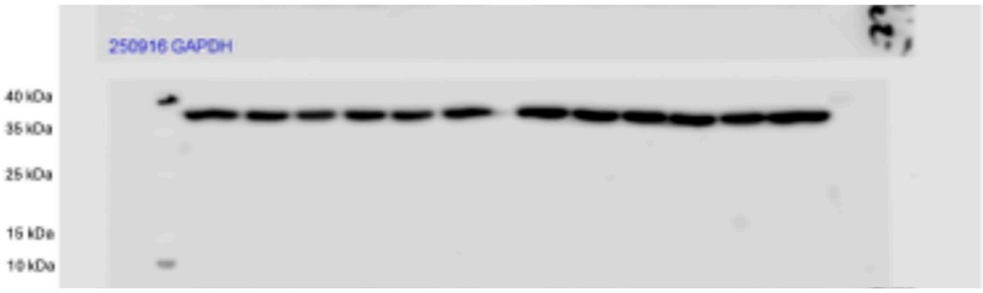

GAPDH loading control, molecular weight: 37 kDa (same samples, 25-09-16).

H2A.X and phospho-H2A.X (Ser139) (with GAPDH loading control)

Note: The protein detected on this blot is labeled "phospho-H2A.X (Ser139)" on the original raw image; it is referred to as p-H2A.X in the main-text Figure 4C. Both terms denote the identical Ser139-phosphorylated epitope of histone H2A.X (also widely abbreviated γH2AX) and are used interchangeably in the literature.

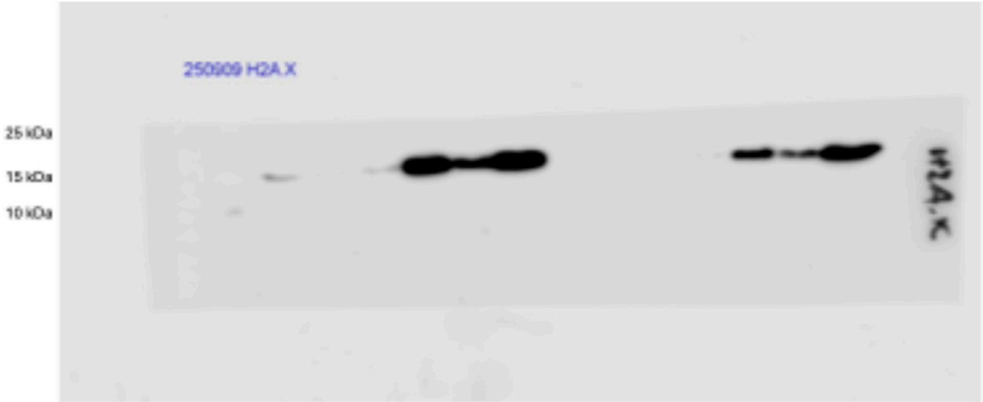

H2A.X, molecular weight: 15 kDa. Experiment 25-09-09.

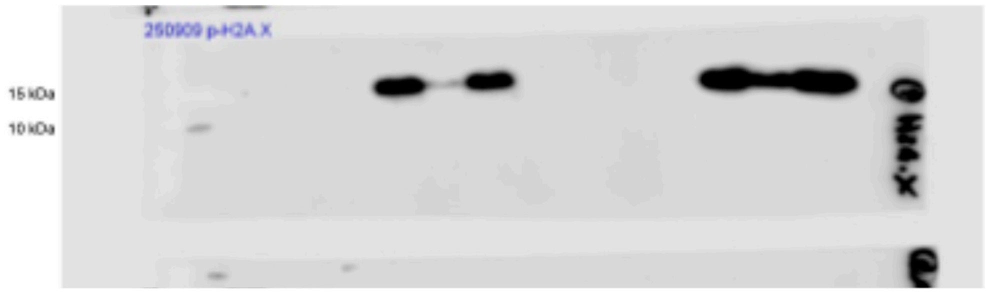

phospho-H2A.X (Ser139), molecular weight: 15 kDa. Experiment 25-09-09.

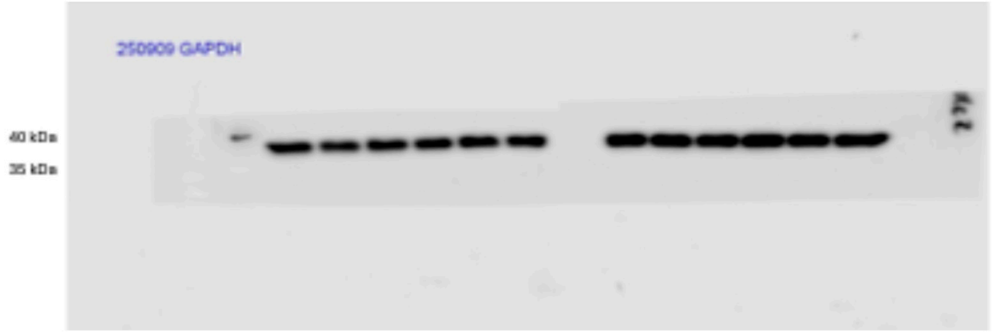

GAPDH loading control, molecular weight: 37 kDa (same samples, 25-09-09)
