# Supplementary material for: WEE1 Inhibition as a Novel Therapeutic Strategy in Cutaneous T-Cell Lymphoma
Source: Cancers (Basel). 2026 Aug 28;18(17):2793. doi: 10.3390/cancers18172793 (PMC13565369; doi:10.3390/cancers18172793)
Supplement: Supplementary file 1 [file cancers-18-02793-s001.zip › Supplementary Table S1.pdf]

Patient ID	Sex	Age, y	CTCL subtype	Stage	B Stage at diagnosis	Aberrant T-cell immunophenotype (clinical / research flow)*	Sorted abnormal cell population (magnetically enriched)§	$CD4^{+}CD7^{-}$, cells/µl, % (clinical)†	$CD4^{+}CD26^{-}$, cells/µl, % (clinical)‡	CD4:CD8 ratio	TCR-$V\beta^{+}$	PCR^{+}	Current therapy	Previous therapies	Most recent prior systemic treatment
**Pt. 1**	m	75	SS	IVA1	B2	$CD4^{+}CD7^{-}CD26^{-}$	$CD4^{+}CD7^{-}$	1830/µl, 24%	21% 1600/µl	15	Yes	Yes	MOGA	UVA1, INF, ECP, Bex, Moga	Bex
**Pt. 2**	m	69	SS	IVA1	B2	$CD4^{+}CD7^{-}CD26^{-}$	CD4+CD7−	4846/µl, 48%	n.a.	11.3	Yes	Yes	-	TCS, systemic steroids	Systemic steroids
**Pt. 3**	f	81	SS	IVA1	B2	$CD4^{+}CD7^{-}CD26^{+}$	CD4+CD7−	627/µl, 75%	n.a.	64.34	Yes	Yes	TCS	NB-UVB, TCS, ECP, Bex, PUVA, MTX, Gem, TCS, ATZ	ATZ
**Pt. 4**	m	59	SS	IVA1	B2	$CD4^{+}CD7^{+}CD26^{-}$	CD4+CD26−	<10/µl, 0.1%	2000/µl, 19%	112	Yes	Yes	Naive	None	Naive

Table S1. Clinical and immunophenotypic characteristics of Sézary syndrome patients included in ex vivo assays. CTCL subtype refers to the subtype at the time of diagnosis. Staging follows the criteria of the International Society for Cutaneous Lymphomas classification. Current therapy is defined as the treatment the patient was receiving at the time of the experiment. $CD4^{+}/CD8^{+}$ ratios and the presence of malignant cells in the blood were assessed by routine clinical flow cytometry, either at the time of the experiment or shortly before. PCR^{+} indicates that ≥1 of 3 PCR reactions identified a clonal T-cell receptor (TCR) gene rearrangement. TCR-$V\beta^{+}$ indicates a dominant usage of a single T-cell receptor Vβ family by flow cytometry. * Summarizes the dominant immunophenotype of the clonal T-cell population in peripheral blood based on routine clinical flow cytometry and, where indicated, additional research flow cytometry including CD26 on ex vivo samples. § Indicates the T-cell fraction used as the malignant population for ex vivo assays, enriched by $CD4^{+}$ T-cell isolation followed by magnetic depletion of the aberrantly absent marker: depletion of $CD7^{+}$ cells in $CD7^{-}$ cases ($CD4^{+}CD7^{-}$ cells) and depletion of $CD26^{+}$ cells in the $CD7^{+}CD26^{-}$ case (patient 4). † Indicates the percentage and absolute number of $CD4^{+}CD7^{-}$ T cells in peripheral blood as determined by routine clinical flow cytometry. ‡ Indicates the percentage and absolute number of $CD4^{+}CD26^{-}$ T cells in peripheral blood from routine clinical flow cytometry. CD26 was available only for patients 1 and 4; in the remaining patients CD26 was not included in the routine clinical panel at the time of sampling. Abbreviations: ATZ, alemtuzumab; BEX, bexarotene; ECP, extracorporeal photopheresis; F, female; Gem, gemcitabine; M, male; MOGA, mogamulizumab; MTX, methotrexate; NB-UVB, narrow band UV-B; PUVA, psoralen plus ultraviolet A; SS, Sézary syndrome; TCS, topical corticosteroids; UVA1, ultraviolet A1.
